# Landscape Changes Influence the Occurrence of the Melioidosis Bacterium *Burkholderia pseudomallei* in Soil in Northern Australia

**DOI:** 10.1371/journal.pntd.0000364

**Published:** 2009-01-20

**Authors:** Mirjam Kaestli, Mark Mayo, Glenda Harrington, Linda Ward, Felicity Watt, Jason V. Hill, Allen C. Cheng, Bart J. Currie

**Affiliations:** 1 Tropical & Emerging Infectious Diseases Division, Menzies School of Health Research, Charles Darwin University, Darwin, Northern Territory, Australia; 2 Department of Natural Resources, Environment and the Arts, Darwin, Northern Territory, Australia; Mahidol University, Thailand

## Abstract

**Background:**

The soil-dwelling saprophyte bacterium *Burkholderia pseudomallei* is the cause of melioidosis, a severe disease of humans and animals in southeast Asia and northern Australia. Despite the detection of *B. pseudomallei* in various soil and water samples from endemic areas, the environmental habitat of *B. pseudomallei* remains unclear.

**Methodology/Principal Findings:**

We performed a large survey in the Darwin area in tropical Australia and screened 809 soil samples for the presence of these bacteria. *B. pseudomallei* were detected by using a recently developed and validated protocol involving soil DNA extraction and real-time PCR targeting the *B. pseudomallei*–specific Type III Secretion System TTS1 gene cluster. Statistical analyses such as multivariable cluster logistic regression and principal component analysis were performed to assess the association of *B. pseudomallei* with environmental factors. The combination of factors describing the habitat of *B. pseudomallei* differed between undisturbed sites and environmentally manipulated areas. At undisturbed sites, the occurrence of *B. pseudomallei* was found to be significantly associated with areas rich in grasses, whereas at environmentally disturbed sites, *B. pseudomallei* was associated with the presence of livestock animals, lower soil pH and different combinations of soil texture and colour.

**Conclusions/Significance:**

This study contributes to the elucidation of environmental factors influencing the occurrence of *B. pseudomallei* and raises concerns that *B. pseudomallei* may spread due to changes in land use.

## Author Summary

Melioidosis is a severe disease affecting humans and animals in the tropics. It is caused by the bacterium *Burkholderia pseudomallei*, which lives in tropical soil and especially occurs in southeast Asia and northern Australia. Despite the recognition that melioidosis is an emerging infectious disease, little is known about the habitat of *B. pseudomallei* in the environment. We performed a survey in the Darwin area in tropical Australia, screening 809 soil samples for the presence of these bacteria using molecular methods. We found that environmental factors describing the habitat of these bacteria differed between environmentally undisturbed and disturbed sites. At undisturbed sites, *B. pseudomallei* was primarily found in close proximity to streams and in grass- and roots-rich areas. In disturbed soil, *B. pseudomallei* was associated with the presence of animals, farming or irrigation. Highest *B. pseudomallei* counts were retrieved from paddocks, pens and kennels holding livestock and dogs. This study contributes to the elucidation of the habitat of *B. pseudomallei* in northern Australia. It also raises concerns that *B. pseudomallei* may spread due to changes in land management.

## Introduction

*Burkholderia pseudomallei* is a Gram-negative bacterium whose main habitat is in moist tropical soil between latitudes 20° N and 20° S [1]. *B. pseudomallei* is not only a soil saprophyte but also a human and animal pathogen causing the severe disease melioidosis [1],[2]. Clinical manifestations range from subclinical infection to localized abscess formation, pneumonia and systemic sepsis with mortality rates up to 90% [3],[4]. A large proportion of melioidosis patients have host predisposing factors such as diabetes, renal disease and alcoholism [5]. The bacteria are mainly transmitted by exposure to contaminated wet soil and surface water and the mode of infection is predominantly percutaneous inoculation, with inhalation and ingestion also reported [6]. Melioidosis is an endemic disease in southeast Asia and tropical Australia. In northeastern Thailand, *B. pseudomallei* accounts for up to 20% of community-acquired septicemia [1] and in Royal Darwin Hospital in northern Australia, melioidosis has been the most common cause of fatal community-acquired bacteremic pneumonia [7].

Despite increased awareness of melioidosis being an emerging disease [6], [8]–[10], not much is known about the habitat of *B. pseudomallei*. Studies have shown that proliferation of *B. pseudomallei* is dependent on high water content of the soil and *B. pseudomallei* has been isolated from muddy, moist and clay-rich soil and pooled surface water [11]–[13]. *B. pseudomallei* has been detected in unchlorinated water supplies [14],[15] and there is a clear positive association between monsoonal rain or extreme weather events and incidence of melioidosis [6],[16]. Environmental studies have shown an association with irrigated cultivated areas such as rice paddies in Thailand [17]–[19] (with corresponding high rates of disease in rice farmers [1],[20]) and anecdotal reports tell of *B. pseudomallei* positive, irrigated sports fields (own observation, [19],[21]). Although this may suggest an association with land use, it is unclear whether this may represent a bias in sampling and exposure as a systematic survey has not been performed.

In order to explore the habitat of *B. pseudomallei* in the tropical Top End of Australia and the influence of environmental manipulations upon its occurrence, we performed a large survey on *B. pseudomallei* occurrence in the soil of the Darwin rural area. Previously, we have developed and validated a molecular tool to detect *B. pseudomallei* in soil [22]. This method was based on soil DNA extraction and real-time PCR and proved to be faster and more sensitive than the gold standard culture, while still being specific [22]. By using this tool, we screened more than 800 soil samples from rural Darwin for the presence of *B. pseudomallei*. With multivariable analyses we discovered new associations between the occurrence of *B. pseudomallei* and environmental factors.

## Materials and Methods

### Soil sampling location and strategies

In the dry season 2006 (July to October, “dry 06”), 499 soil samples were collected at a depth of 30 cm from 141 soil sampling sites within a 50 km radius of Darwin (12° S) in the Top End of the Northern Territory in Australia. The Top End mainly consists of tropical savanna [23] and wetland ecosystems [24]. Sites were randomly chosen using the “Random Point Generator” extension for ArcView 3.2. with the following restrictions: The Darwin rural region was subdivided into nine rural areas (average area 51 km^2^, SD 12 km^2^) and within each area, sites were distributed between undisturbed and environmentally manipulated sites. The latter consisted of residential or farming properties with livestock (horses, cattle, pigs or chickens) or fruit farming (predominantly mango farms). Four samples were collected per undisturbed or farm site and two samples per residential property. In the wet season 2007 (March to April, “wet 07”), 74 of the 141 sites were visited again and another 256 soil samples were collected at a depth of 30 cm (see Table 1). These 74 sites consisted of all accessible, previously positive sites (30 of 38 previously positive sites) and 44 controls from the same rural areas. Sites were matched for level of environmental manipulation and waterlogging. In the dry season 2007 (July to September, “dry 07”), 30 sites were visited again of which 17 were previously positive following the same scheme as above and 54 soil samples were collected. Soil was collected into sterile 50 mL specimen containers containing 5 ml of dH_2_O and auger and spade were cleaned with 70% ethanol between soil collections. Soil sampling sites were mapped and various environmental factors were recorded on site such as distance to next stream, vegetation class, presence of roots in soil, presence of animals (livestock, dogs or native animals such as wallabies; the latter was declared positive if droppings were sighted in close proximity of the sampling site). Soil water status and soil texture were determined using the “Australian Soil and Land Survey” Field Handbook [25] and by following a common soil texture flowchart (http://www.h2ou.com/h2twss96.htm). Soil color was interpreted using the Munsell Soil Color Chart as described previously [22].

**Table 1:** *B. pseudomallei* screening results from soil sampling at 30 cm depth.

		Number of Sites	*% B.ps* Positive Sites (n)	Number of Samples	*% B.ps* Positive Samples (n)
**Dry season 2006**	**Sites / Samples**	**141**	**27% (38)**	**499**	**11% (57)**
	Undisturbed	53	21% (11)	210	11% (23)
	Residential	32	31% (10)	65	17% (11)
	Farm	56	30% (17)	224	10% (23)
**Wet season 2007**	**Sites / Samples**	**74**	**35% (26)**	**256**	**16% (41)**
	Undisturbed	26	46% (12)	104	20% (21)
	Residential	20	20% (4)	40	10% (4)
	Farm	28	36% (10)	112	14% (16)
**Dry season 2007**	**Sites / Samples**	**30**	**33% (9)**	**54**	**17% (9)**
	Undisturbed	11	17% (3)	18	17% (3)
	Residential	9	22% (2)	12	17% (2)
	Farm	10	40% (4)	24	17% (4)
	**Total**	**141**	**34% (48)**	**809**	**13% (107)**

Site and sample distribution with *B. pseudomallei* (*B.ps*) screening results. 809 soil samples were collected in the Darwin rural area in the dry season of 2006 and the following wet and dry season of 2007. 41% and 57% of re-visited sites in the wet season 2007 and dry season 2007 were positive in the previous dry season 2006.

### Soil DNA extraction

DNA was extracted from 20 g of soil as previously described [22]. While 20 g of most soils roughly equaled 20 mL of volume, a few soils showed large mass variations. We therefore set a lower and upper volume limit for all soil samples of 15 to 25 mL to avoid large variations in volume. In brief, starting with an enrichment step incubating 20 g of soil in 20 mL of selective modified Ashdown's broth [26] for 39 hours shaking at 37°C, 1 mL of CaCO_3_ saturated water was added and the sample was centrifuged twice. The soil pellet was processed for DNA extraction using a modified protocol of the ultraclean soil DNA isolation kit (MoBio Laboratories, USA). Modifications included the addition of 0.8 mg of aurintricarboxylic acid (ATA) and 20 µL of proteinase K (20 mg / mL). DNA was purified further with the QIAamp DNA Micro Kit (Qiagen, Hilden, Germany) and eluted in 50 µL of 10 mM Tris HCl, 0.5 mM EDTA, pH 9.0.

### Detection of *B. pseudomallei* DNA by TTS1 real-time PCR

*B. pseudomallei* DNA was detected by real-time PCR using a Rotor-Gene 2000 (Corbett Research, Australia) targeting a 115 bp stretch of the *B. pseudomallei* specific *orf2* of type III secretion system (TTS1) as described in [22]. Briefly, 4 µL of DNA were amplified in duplicates in 25 µL volumes. The probe was at a final concentration of 256 nM and labelled with FAM and a black hole quencher (Biosearch Techonologies). Supplementary reagents included 0.25 U Uracil DNA Glycosylase (Invitrogen), dUTPs and nonacetylated BSA at final 400 ng / µL. Non-Template Controls (NTC) were added to each run and no amplifications were detected. In order to check for PCR inhibitors, 0.3 pg of an inhibitor control plasmid [22] were amplified alone and in parallel spiked with 4 µL of sample DNA. In each PCR run, the plasmid was also used as standard positive control in a dilution series in duplicates and at final concentrations of 4.4 ng / mL, 217 pg / mL, and 11 pg / mL. Ct values had an average of 33.2 with a 95% confidence interval (95% CI) of 32.3–34.1.

### Statistical analysis

Statistical analysis was carried out using Stata (Intercooled Stata, version 8.2, USA). For univariate analysis, Fisher's Exact test and Mann-Whitney U test were used. For multivariable analysis, odds ratios were calculated in stepwise multivariable logistic regression analyses clustered by site. The specification of the models was assessed using a link test. All tests were 2-tailed and considered significant if P values were less than 0.05.

Autocorrelation was assessed by calculating Geary's c statistic, which tests the null hypothesis of global spatial independence (indicated by values of around 1), in bands of 0.05 degrees (5.6 km) up to 0.20 degrees (22.2 km). We performed all further analysis assuming spatial independence where there was no evidence of significant autocorrelation.

One-way Analysis of Similarities (ANOSIM) is a non-parametric permutation procedure (999 permutations) and was used to test the null hypothesis of no difference in the composition of environmental variables between two groups of soil samples (for instance, *B. pseudomallei* positive versus negative soil samples); it was based on a resemblance matrix of Euclidean distances between soil samples with short distances indicating high similarities between the composition of environmental variables of two soil samples (normalized data). Similarity Percentage Breakdown (SIMPER) analysis was used to evaluate the main environmental factors which were responsible for the observed clustering of samples (also using a Euclidean Distance matrix with normalized data). Principal Component Analysis (PCA) proved useful to visualize the dataset based on the combination of environmental factors describing the soil samples. The normalized dataset was projected onto a 2-dimensional ordination with the axes maximizing the variance of the data. The axes are a linear combination of environmental factors and the vectors reflect the coefficients of these factors. ANOSIM, SIMPER and PCA analyses were performed using Primer 6.1.9 (Primer-E Ltd., UK).

## Results

### Detection of *B. pseudomallei* in soil

**Figure 1:**
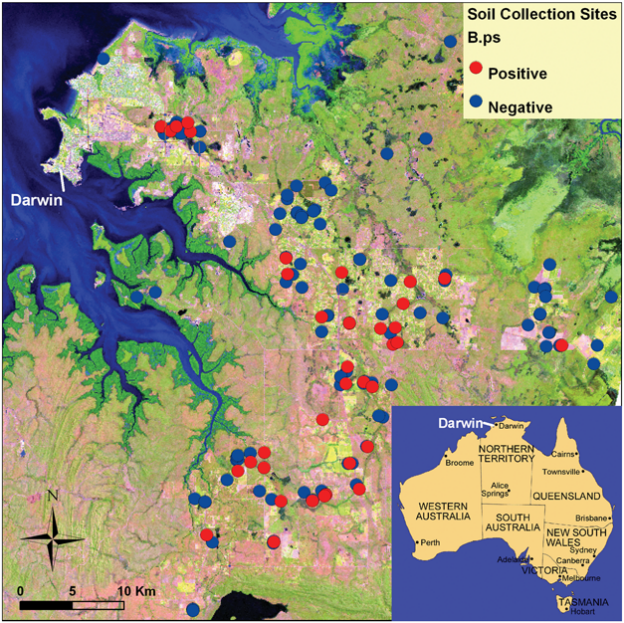
Map of rural Darwin. Map of rural Darwin showing soil sampling sites with red dots indicating *B. pseudomallei* positive sites in the dry season 2006 and blue dots no detection of *B. pseudomallei*. Inset shows map of Australia.

809 soil samples collected at a depth of 30 cm were screened for *B. pseudomallei* by using a previously developed and validated soil DNA extraction and real-time PCR protocol [22]. Screening resulted in a total of 107 *B. pseudomallei* positive samples from 48 sites (see Table 1, 2 and Figure 1). In the dry season 2006, soil samples of 21% (11 / 53) of undisturbed and 31% (27 / 88) of environmentally disturbed sites such as farms or residential properties tested positive for *B. pseudomallei* (Fisher's Exact, P = 0.242).

**Table 2:** Comparison of *B. pseudomallei* screening results between dry and wet season.

Repeated sampling at same sites in dry 06 and wet 07			
Category	Dry & Wet Positive	Dry only Positive	Wet only Positive
Undisturbed	11% (11/104)	7% (7/104)	10% (10/104)
Residential	8% (3/40)	13% (5/40)	3% (1/40)
Farm	9% (10/112)	6% (7/112)	5% (6/112)

*B. pseudomallei* screening results were compared between the dry season 2006 and following wet season 2007. % refers to *B. pseudomallei* positive samples as a proportion of total soil samples collected in the corresponding category during that time.

**Figure 2:**
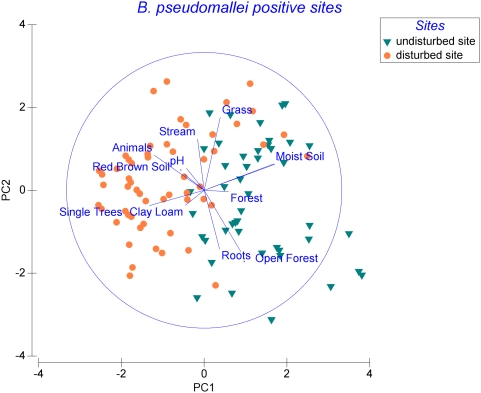
Principal Component Analysis (PCA) of *B. pseudomallei* positive soil samples. Green triangles refer to soil samples of undisturbed sites whereas orange dots are samples of environmentally manipulated areas. The axes of the PCA ordination plot are a linear combination of environmental factors describing the soil samples and the vectors reflect the coefficients of these factors indicating the direction and strength of the correlation. The maximum possible strength of all correlations is indicated by the blue circle. Explained variance - PC1 axes: 20.2%; PC2 axes: 13.3%. The first 5 principal components accounted for 67% of the observed variance in the dataset.

Various environmental factors such as soil texture, soil colour, soil moisture, vegetation class, presence of animals and distance to a stream were recorded for all soil samples. In a multivariable logistic regression analysis, significant risk factors for the presence of *B. pseudomallei* were close proximity to a stream (Odds Ratio OR 2.5, 95% CI 1.3–4.9), moist soil (OR 2.6, 95% CI 1.6–4.2), the presence of animals (OR 2.4, 95% CI 1.3–4.6), as well as roots-rich soil (OR 1.8, 95% CI 1.1–3.0) and red brown - grey soil (OR 3.4, 95% CI 1.4–8.0; OR 2.8, 95% CI 1.1–6.9) (see also Table 3). In a Principal Component Analysis (PCA), the composition of environmental factors was compared between all *B. pseudomallei* positive soil samples and a separate clustering was evident for soil samples collected at undisturbed sites as compared to environmentally manipulated sites (see Figure 2). This was also confirmed by a non-parametric permutation procedure called Analysis of Similarities (ANOSIM) (P = 0.001). The vectors in Figure 2 show that *B. pseudomallei* positive undisturbed sites exceed disturbed sites in waterlogged and roots-rich soil, (open) forests such as found along creeks whereas environmentally disturbed *B. pseudomallei* positive sites had a higher proportion of animal resting places, red brown soil, clay loam or single trees such as found on mango farms and paddocks. This was consistent with a Similarity Percentage Breakdown (SIMPER) analysis which evaluates the main environmental factors responsible for the observed clustering and showed a similar contribution of factors to the clustering as PCA. Both SIMPER and PCA analysis also revealed that *B. pseudomallei* positive native sites exceeded positive disturbed sites in moist soil. Indeed, significantly more *B. pseudomallei* positive soil samples were classified as dry if from disturbed areas (37% dry (29/78)), especially from non-irrigated disturbed sites (56% dry (13/24)) as compared to undisturbed sites (17% dry (10/58)) (Fisher's Exact, P = 0.013 and P = 0.001 for non-irrigated sites). Of the 24 *B. pseudomallei* positive soil samples from non-irrigated disturbed sites, 16 (67%) were collected in close proximity to or within pens, paddocks or kennels with livestock, chickens or dogs.

**Table 3:** *B. pseudomallei* occurrence and environmental factors.

Environmental Factors	Multivariable Logistic Regression Models OR (95% CI) P value		
	Model Undisturbed	Model Disturbed	Model Overall
Distance to Stream <10 m	**8.6** (2.6–28.4) 0.000		**2.5** (1.3–4.9) 0.008
Moist Soil	**4.2** (1.9–9.6) 0.001		**2.6** (1.6–4.2) 0.000
Animals	**3.6** (1.4–9.3) 0.008	**3.8** (1.7–8.2) 0.001	**2.4** (1.3–4.6) 0.006
Spear Grass	**5.2** (1.7–16.1) 0.004		
Roots	**3.1** (1.4–6.9) 0.007		**1.8** (1.1–3.0) 0.016
Clay Loam		**3.1** (1.2–8.1) 0.021	
Red Brown Soil	**2.1** (1.0–4.3) 0.038		**3.4** (1.4–8.0) 0.006
Red Grey Soil			**2.8** (1.1–6.9) 0.024
IA Clay+Red Brown Soil		**2.3** (1.4–3.9) 0.002	

Multivariable logistic regression analysis of environmental factors contributing to the presence of *B. pseudomallei* in soil at either undisturbed, disturbed sites or overall. The analysis was clustered for sites and all odds ratios (OR) were statistically significant. All models were specified correctly as tested by a linktest. “IA Clay+Red Brown Soil” refers to interaction between clay and the soil color red brown.

#### *B. pseudomallei* in soil of undisturbed sites

At environmentally undisturbed sites, a strong association between *B. pseudomallei* and grass and roots-rich soil was evident (Fisher's Exact, P<0.001 and P = 0.001 respectively). This was confirmed by multivariable logistic regression analysis with grass such as spear grass (e.g. *Sorghum spp.*) being a significant contributor to the model (see Table 3). In the dry season, uni- and multivariable analysis showed a significant association between *B. pseudomallei* and resting places of native animals such as wallabies (Fisher's Exact, P<0.001)(see Table 3). Further risk factors for the presence of *B. pseudomallei* at undisturbed sites were close proximity to a stream; moist soil and red brown soil (see Table 3).

Sampling repeated in the wet season 2007 showed a proportional increase of *B. pseudomallei* positive samples from undisturbed sites (Fisher's Exact, P = 0.009) in the wet season as compared to the dry 2006, which was in contrast to environmentally manipulated sites where no such increase was evident (Fisher's Exact, P = 0.259)(see Table 2).

#### *B. pseudomallei* in soil of environmentally manipulated sites

At disturbed sites, the presence of *B. pseudomallei* was significantly associated with clay loam (Fisher's Exact, P = 0.013). This soil texture was mainly found on environmentally manipulated sites (Fisher's Exact, P<0.001) and in association with roots-rich soil (Fisher's Exact, P<0.001). In multivariable logistic regression analysis, significant risk factors for the presence of *B. pseudomallei* at environmentally manipulated sites were the presence of animals (livestock, dogs, wallabies), the soil texture clay loam as well as red brown clay (see Table 3).

In the dry season, gardens of residential properties showed a higher prevalence of *B. pseudomallei* as compared to other sites (Fisher's exact, P = 0.035), which was reversed in the wet season (see Table 2). In the dry season, *B. pseudomallei* was more often found in lawn areas as compared to garden beds (Fisher's Exact, P = 0.006) and *B. pseudomallei* positive garden soil showed significantly lower pH (positive garden soil: median pH 5.5, bootstrap estimate 95% CI 5.2–5.8; negative garden soil: median pH 6.5, 95% CI 6.0–7.0) (Mann Whitney U test, P = 0.008).

### Prediction of areas for presence of *B. pseudomallei*

The combination of environmental factors describing *B. pseudomallei* positive undisturbed sites is a clear-cut subset of factors describing all samples collected at undisturbed sites (ANOSIM, P = 0.003). For soil samples collected at undisturbed sites, SIMPER analysis showed that more than 50% of the observed clustering of *B. pseudomallei* positive samples versus negative samples was due to a higher percentage of positive soil samples being moist, red brown or roots-rich and having been collected at grass-rich sites and in forests (the latter mainly along creeks). This distinct environmental factor composition of positive versus negative samples was used to explain different *B. pseudomallei* prevalence rates in different areas of rural Darwin. For instance, one area showed a significantly lower *B. pseudomallei* occurrence compared with another area (1.6% versus 21.9%, Fisher's Exact, P<0.001). This matched with significantly more sandy soil samples (34.7% versus 12.9%, Fisher's Exact, P<0.001) and less sampling sites rich in spear grass (0% versus 12.9%, Fisher's Exact, P<0.001) in the area of lower *B. pseudomallei* occurrence.

In order to assess whether the *B. pseudomallei* status of undisturbed sites clustered together, an autocorrelation analysis was performed. Using both positive (n = 23) and negative (n = 187) points, there was some evidence of negative autocorrelation in the 0–0.05 degree (5.6 km) band (Geary's c 1.17, p = 0.015) indicating weak evidence of a negative correlation between positives and negatives within this band. A positive autocorrelation was detected in the 0.05–0.10 degree (5.6–11.1 km) band (Geary's c 0.88, p = 0.034) which matches the above finding of different *B. pseudomallei* prevalence rates in different areas of rural Darwin. Because the magnitude of autocorrelation was weak and inconsistent between bands, we performed further analysis assuming spatial independence.

No autocorrelation was detected for environmentally disturbed areas and no specific factor combination was evident for *B. pseudomallei* positive environmentally disturbed areas apart from the presence of livestock and pets, impeding the prediction of *B. pseudomallei* occurrence at environmentally manipulated sites.

## Discussion

The major finding of this study is that two sets of environmental factors describe the habitat of *B. pseudomallei* in the Top End. One set refers to the habitat of *B. pseudomallei* at undisturbed sites whereas the other one characterises environmentally manipulated sites.

At undisturbed sites, *B. pseudomallei* was frequently found along creeks in highly vegetated areas. *B. pseudomallei* positive sites were often in close proximity to annual spear grass (such as *Sorghum spp.*) and grasses in riparian zones. Some of these grasses are known to have an extensive root system reaching down to the ground water to survive in the dry season [27], which would be a favorable feature for the survival of water dependent *B. pseudomallei* in the dry season. In the wet season, we also observed a strong increase of *B. pseudomallei* load at sites rich in spear grass (data not shown), which coincides with the time when annual grasses flourish involving a high increase of fine root mass [28]. Data on *B. pseudomallei* load was obtained by an approximate semi-quantification method measuring soil *B. pseudomallei* load from standard curves generated in soil inoculation experiments [22] and the inclusion of an internal plasmid into the soil DNA extraction as additional efficiency control. However, this quantification data should be interpreted with caution because of only moderate reproducibility. Work is ongoing in validating these preliminary results and determining their significance. Current data suggest that *B. pseudomallei* might be associated with roots of some of these grasses. This would not be surprising as many relatives of *B. pseudomallei* such as those of the *B. cepacia* genomovars are closely associated with the rhizosphere i.e. with the soil immediately surrounding the roots of plants [29]–[31]. Our current results do not allow any conclusions on which grass species in particular were associated with *B. pseudomallei*. This will be addressed in future field and *in vitro* studies.

*B. pseudomallei* could also be associated with arbuscular mycorrhizal fungi (AMF) which are symbionts of many plants and also live in the rhizosphere. *B. pseudomallei* has been shown *in vitro* to be able to colonize spores of AMF such as *Gigaspora decipiens* [32].

At environmentally manipulated sites, highest *B. pseudomallei* counts were retrieved from paddocks, pens and kennels holding horses, pigs, chickens or dogs and cats (data not shown). We also found significantly more *B. pseudomallei* positive soil samples which were dry in environmentally manipulated areas as opposed to undisturbed sites. This suggests that other factors make up for the reduced water supply while the observed high *B. pseudomallei* counts may indicate superior growth conditions for *B. pseudomallei* at some disturbed sites. The observed strong association of *B. pseudomallei* with the presence of animals raises the question whether these animals were infected with *B. pseudomallei* and acted as an amplification stage for these bacteria. We cannot rule out this possibility. However *B. pseudomallei* is highly pathogenic for most farm animals and asymptomatic carriage has generally only been reported in pigs [2]. Thus, more likely explanations are digging or foraging activities of animals increasing soil aeration [33] and water infiltration [34] or increased access to organic material and nitrogen derived from animal waste. All this could contribute to the growth of the preferentially aerobic saprophyte, together with soil acidification processes which are a by-product of nitrification processes [35]–[37]. We observed a significantly lower pH of *B. pseudomallei* positive soils. This was mainly evident in garden soil where the pH was generally in a more neutral range than for other soils studied. In contrast, the pH range of undisturbed soil overlapped the pH of most *B. pseudomallei* positive soils.

Less *B. pseudomallei* positive soil samples were retrieved from residential properties in the wet season as opposed to the dry season, which was in stark contrast to undisturbed sites where *B. pseudomallei* prevalence increased in the wet. We hypothesize that on residential properties, *B. pseudomallei* might be spread by irrigation systems of gardens which are only operated in the dry season. Up to 33% of water bores of rural residential properties are *B. pseudomallei* positive (Mark Mayo, manuscript in preparation) and irrigation systems are fed by these bores. Hence, not only might irrigation of gardens and cultivated areas improve surface conditions for the survival of *B. pseudomallei*, but irrigation systems themselves might also actively pump bacteria to the surface.

Our data clearly indicate that environmental perturbations have an influence upon the occurrence of *B. pseudomallei*. Land use management such as agriculture has been shown to have a large effect on soil bacteria and their community structure [38]. Bacterial diversity was shown to decrease on arable land [39] and a shift to *Burkholderia spp.* was evident after a change from forest to pasture vegetation in one study [40]. An increase of *B. pseudomallei* load on farm properties could lead to melioidosis outbreaks in livestock such as goats, sheep or pigs which have been reported [2] including in non-endemic areas [41]. Further soil perturbations such as those caused by construction and soil excavation work have been associated with a melioidosis outbreak in Western Australia in the dry season [42]. Also extreme natural events such as monsoonal heavy rains, cyclones or tsunamis have a large impact upon landscapes and soil and such events have been reported to be associated with an increase of melioidosis incidence [16], [43]–[45]. Sporadic flooding has also unmasked melioidosis in areas such as temperate southern Queensland, despite this region being considerably south of the melioidosis endemic belt in tropical Australia [46]. Our data also suggest that in other endemic regions of the world such as Thailand, agricultural practices like rice farming may favor the growth of *B. pseudomallei* and might contribute to the strong association of *B. pseudomallei* with rice fields observed in southeast Asia [17]–[19]. However other factors including different soil, vegetation and climate, the nature of soil disturbance and the interaction with different environmental microbes including the closely related *B. thailandensis*, limit the generalizability. Further studies are therefore required in other regions with different environmental conditions.

Whereas clay was only significantly associated with *B. pseudomallei* in combination with the soil color red brown (indicating oxidized iron), a strong correlation between clay loam and *B. pseudomallei* was evident. Clay loam is roughly an equal mixture of clay, silt and sand [25]. While clay provides excellent water and nutrient retention abilities due to its large surface area and chemical activity, clay loam is less dense than clay, less prone to waterlogging and provides better aeration for e.g. plant root development which is why, clay loam is often regarded as a good garden soil. Therefore, it was no surprise to find a significant association with clay loam and roots-rich soil. However, clay loam is not a typical soil of the Top End and was mainly found on environmentally manipulated sites such as farms or gardens. Common soil types in rural Darwin are kandosols which are well drained, gravelly, yellow or red massive earths often overlaying weathered, iron-rich material [47]. Along drainage lines and creeks, hydrosols are common, which are seasonally wet, sandy massive earths. A widespread topsoil of the Top End is sandy loam over sandy clay loam subsoil and light to medium clay at depth.

In the last two decades, there has been a substantial increase of human activities in the Darwin rural area where this study was undertaken. In particular, small scale horticulture and farming have expanded on the many rural land blocks. This has not only increased the number of people being exposed to *B. pseudomallei* but *B. pseudomallei* itself could potentially be spreading along with the ongoing land management changes. There are also on-going changes in landscape ecology in northern Australia inflicted by changed fire regimes which led to an increase of some annual grasses [48] and the introduction of invasive plant species such as *Andropogon gayanus* Kunth (Gamba grass). These changes have a large impact on native grasses, soil moisture and soil nitrogen cycles [49],[50] which could further prove advantageous to the survival of *B. pseudomallei* such as the potential spread of grasses associated with *B. pseudomallei* or the persistence of annual soil wetting caused by invasive wetland grasses [51].

In summary, we have described a combination of environmental factors that are strongly associated with the presence of *B. pseudomallei* in the tropical Top End of Australia and we provide evidence that changes in land use influence the occurrence of *B. pseudomallei*. Therefore, melioidosis and *B. pseudomallei* might not only be an emerging infectious disease due to improved recognition and diagnostic techniques [8], but they might indeed be spreading in and beyond endemic locations because of complex environmental disturbances and changed landscape ecology.
